# Factors and reasons for planning to quit smoking among a nationally representative sample of adults who smoke: Findings from the 2021 ITC EUREST-PLUS Spain Survey

**DOI:** 10.18332/tpc/192088

**Published:** 2024-11-20

**Authors:** Susan C. Kaai, Marcela Fu, Pete Driezen, Anne C. K. Quah, Mi Yan, Yolanda Castellano, Olena Tigova, Geoffrey T. Fong, Esteve Fernández

**Affiliations:** 1 Department of Psychology, University of Waterloo, Waterloo, Canada; 2 School of Public Health Sciences, University of Waterloo, Waterloo, Canada; 3 Tobacco Control Unit, Catalan Institute of Oncology – WHO Collaborating Centre for Tobacco Control, l’Hospitalet de Llobregat, Barcelona, Spain; 4 Tobacco Control Research Group, Bellvitge Biomedical Research Institute, l’Hospitalet de Llobregat, Barcelona, Spain; 5 Department of Public Health, Mental Health, and Maternal and Child Health Nursing, School of Nursing - Bellvitge Campus, University of Barcelona, Barcelona, Spain; 6 Center for Biomedical Research in Respiratory Diseases, Institute of Health Carlos III, Madrid, Spain; 7 Department of Clinical Sciences, School of Medicine and Health Sciences - Bellvitge Campus, University of Barcelona, Barcelona, Spain; 8 Ontario Institute for Cancer Research, Toronto, Canada

**Keywords:** global health, smoking cessation, quit intentions, Spain, cigarettes

## Abstract

**INTRODUCTION:**

Intentions to quit are the strongest predictor of successful smoking cessation and future quit attempts. This study assesses factors associated with quit intentions among adults who smoke in Spain.

**METHODS:**

Data are from the 2021 International Tobacco Control (ITC) EUREST-PLUS Spain Wave 3 Survey, a nationally representative survey of adults aged ≥18 years who smoke (n=1006). Analysis was restricted to 867 adults who provided information about quit intentions. Multivariable Poisson regression was used to examine several correlates of quit intentions. Adjusted prevalence ratios (APR) were estimated.

**RESULTS:**

Less than half (45.6%) of adults who smoke reported intending to quit, with only 13.0% intending to quit in the next 6 months; 11.3% reported at least one quit attempt in the past year. Factors associated with quit intentions were having a high income (APR=1.39; 95% CI: 1.01–1.92), having at least one quit attempt in the previous year (APR=1.41; 95% CI: 1.16–1.71), worrying that smoking will damage one’s health (APR=1.52; 95% CI: 1.05–2.20), regretting starting to smoke (agree, APR=1.25; 95% CI: 1.03–1.52; disagree, APR=0.66; 95% CI: 0.46–0.95), health concerns (APR=1.46; 95% CI: 1.17–1.82), and smoking restrictions in public places (APR=1.28; 95% CI: 1.06–1.54).

**CONCLUSIONS:**

Only13% of adults from Spain who smoke intend to quit in the next 6 months. Factors associated with quitting were high income, at least one quit attempt in the past year, worrying about health damage from smoking, regretting starting to smoke, having health concerns, and smoking restrictions in public places. There is a need for comprehensive measures that encourage and support people to quit.

## INTRODUCTION

Globally, tobacco use is the leading cause of preventable disease, disability, and death. It kills 8 million people annually, including 1.3 million people who do not smoke but are exposed to secondhand smoke. In 2022, 22% of adults from Spain reported smoking; 20% smoked daily^1^. Based on data from the 1987 National Health Survey (ENSE) and the 2020 European Health Survey in Spain (EESE), tobacco use prevalence in Spain’s adult population has slowly decreased^2^. However, the smoking burden remains high, with 53800 to 69000 deaths attributable to smoking each year in Spain^3^. Smoking cessation remains a national priority.

The WHO Framework Convention on Tobacco Control (WHO FCTC) is an evidence-based global health treaty developed in response to the tobacco epidemic^4^. Spain became a Party to the WHO FCTC on 16 June 2003 and ratified the treaty on 11 January 2005^4^. Article 14 of the WHO FCTC obligates Parties to implement effective measures to promote tobacco cessation and provide adequate treatment for tobacco dependence^4^. Several Spanish regions have quit lines for people seeking support to quit. Moreover, in both primary and hospital care, nicotine replacement therapy and/or some cessation services are provided^5^.

Increasing cessation rates among people who smoke is key to reducing smoking-related morbidity and mortality in Spain. The smoking cessation process has been explained using different behavioral theories including the Theory of Planned Behaviour (TPB)^6^ and the Transtheoretical Model of Change (TTM)^7^. Although TPB and TTM differ in their theoretical foundations and in the constructs they include, both models include intentions to quit smoking as a critically important variable, and empirical studies have confirmed this: intentions to quit smoking are known to be among the strongest predictors of future quit attempts and successful smoking cessation^8^. Because intentions to quit smoking are such a strong predictor of quit attempts and successful quitting, it is critically important to identify and understand the factors associated with quit intentions to provide insights into how those intentions might be strengthened to increase quit attempts as well as what factors seem to inhibit those who smoke from attempting to quit.

Past research has found that quit intentions are associated with a number of factors including sociodemographics^9-12^, smoking-related behavior (e.g. nicotine dependence, past quit attempts and receiving quit advice)^11-15^, health concerns^11-15^, regretting starting smoking^15,16^, attitudes and perceptions (e.g. beliefs about health effects of smoking and quitting)^11,14^, knowledge of health risks^17,18^, and smoking restrictions in public venues^19^. This study assessed the extent to which these factors predict quit intentions among adults who smoke in Spain. Although a few studies from Spain have examined factors associated with quit intentions, most of these studies have used smaller sample sizes from specific populations^20-22^; however, our study is nationally representative and uses standardized measures associated with quit intentions, which have been used in 30 countries participating in the ITC Project, allowing comparisons to be made across countries.

## METHODS

### Study design and sample

Data came from the 2021 International Tobacco Control (ITC) EUREST-PLUS Spain Wave 3 Survey, a nationally representative survey of adults who smoke (n=1006), aged ≥18 years. The ITC EUREST-PLUS Spain Wave 3 (2021) Survey is a follow-up survey of the ITC EUREST-PLUS Wave 2 Survey (2018). The Wave 3 survey was conducted from 9 June to 5 August 2021 using computer-assisted personal interviewing or a modified computer-assisted telephone interview for respondents preferring to be interviewed by telephone due to concerns about COVID-19^23,24^. Analysis was restricted to the adults who smoked at least monthly and answered the questions on quit intentions (n=847).

The ITC EUREST-PLUS Spain Project received ethics approval from the Research Ethics Board at the Bellvitge University Hospital in Spain (PR248/17) and the University of Waterloo in Canada (REB#41105). All respondents received information on data confidentiality, security, the potential risks, and benefits of their participation and gave consent to participate.

### Measures

*Dependent variable: Intentions to quit smoking*

Intentions to quit was measured using the following question: ‘Are you planning to quit smoking?’. Respondents who answered ‘in the next month’, ‘in the next 6 months’, or ‘sometime in the future, beyond 6 months’ were classified as intending to quit whereas those who responded ‘not planning to quit’ were classified as not intending to quit. ‘Don't know’ responses were set to missing (n=17).

*Independent variables*

The sociodemographic variables used in this study were: 1) sex (male, female); 2) age at the time of the survey (18–24, 25–39, 40–54, and ≥55 years); 3) monthly household income (€) (low: <1250, moderate: 1250 to <2000, high: ≥2000, not stated); and 4) highest level of education (low: ≤ lower secondary, moderate: upper secondary to some college/university, high: completed university/postgraduate).

Smoking behaviors assessed were smoking status (non-daily, daily), any attempt to quit in the past year (yes, no), and cigarettes smoked per day (1–10, 11–20, ≥21). Nicotine dependence was measured using the Heaviness of Smoking Index (HSI 7 levels: 0=lowest level of addiction to 6=highest level of addiction), defined as the sum of the number of cigarettes smoked per day (‘0’=1–10; ‘1’=11–20; ‘2’=21–30; and ‘3’ ≥31), and time to first cigarette of the day (‘0’ >60 min, ‘1’=31–60 min, ‘2’=6–30 min, ‘3’ ≤5 min)^25^.

Two motivational variables were assessed. The first was worry that smoking will damage health; responses were dichotomized to ‘not at all worried’ and ‘a little/moderately/very worried’. ‘Don't know’ and ‘refused’ responses were set to missing (n=13). The second was on concern that smoking has damaged health; responses were dichotomized to ‘not at all’ and ‘just a little/a fair amount/a great deal’. ‘Don't know’ and ‘refused’ responses were set to missing (n=67).

Regret over starting to smoke was assessed by rating of agreement with the statement: ‘If you had to do it over again, you would not have started smoking’. This variable was recoded into three categories: agree (‘agree/strongly agree’), disagree (‘disagree/strongly disagree’), and neutral (‘neither agree nor disagree)’. ‘Don't know’ and ‘refused’ responses were set to missing (n=18).

Finally, reasons that led respondents to think about quitting were assessed by rating of agreement with the statement: ‘In the past 6 months, have each of the following things led you to think about quitting: 1) concern for health; 2) advice from a doctor, dentist, or other health professional to quit; and 3) smoking restrictions in public places like restaurants, cafés and pubs’. Responses to these three items were dichotomized (‘not at all’ and ‘somewhat/very much’). ‘Don't know’ and ‘refused’ responses were set to missing, i.e. ‘concern for health’ as a reason to quit (n=10), ‘advice from health professional’ (n=2), and ‘smoking restrictions’ (n=1).

A ‘wave of recruitment’ variable was constructed to represent the number of times a respondent participated in the survey to account for potential differences in individuals’ responses between those who were newly recruited compared with those who participated in one or more prior survey waves^26^.

### Statistical analyses

Data were analyzed using SUDAAN (version 11.0.3) to account for the multistage sampling design and sampling weights. Multivariable Poisson regression models were estimated to examine associations between the independent variables and intentions to quit. Models were also used to estimate the predicted marginal probability of intending to quit, or adjusted percentages, and adjusted prevalence ratios (APR)^27^. The variables were adjusted for sex, age, income, and education level. Bootstrap replicate weights were used for variance estimation (500 bootstrap weights, ADJFAY=12 in SUDAAN, denominator degrees of freedom=80). Models also adjusted for the survey wave of recruitment to account for the number of times a respondent previously participated in the survey.

## RESULTS

Table 1 shows the sociodemographic characteristics of the study sample. On average, these adults were aged 45 years, 50.5% male, and 10.6% had a high level of education. Table 2 presents weighted estimates for adults from Spain who smoke: 11.3% had made at least one quit attempt in the past year and less than half (45.6%) reported they intended to quit smoking at any point in the future. Only 13.0% reported intending to quit in the next 6 months.

**Table 1:** Sociodemographic characteristics of a nationally representative sample of adults who smoke from the ITC EUREST-PLUS Spain Survey, Spain, 2021 (N=864)

*Characteristics*	*Unweighted*		*Weighted*	
	*n*	*%*	*%*	*95 % CI*
**Wave of recruitment^a^**				
Wave 1 (2016)	311	36.0	37.0	32.3–42.0
Wave 2 (2018)	117	13.5	12.5	9.8–15.8
Wave 3 (2021)	436	50.5	50.5	45.5–55.5
**Sex**				
Male	464	53.7	50.5	46.9–54.1
Female	400	46.3	49.5	45.9–53.1
**Age** (years)				
18–24	65	7.5	8.6	6.8–11.0
25–39	246	28.5	27.6	24.4–31.2
40–54	316	36.6	31.5	28.1–35.1
≥55	237	27.4	32.2	28.4–36.3
**Income level**				
Low	186	21.5	22.4	18.4–27.0
Moderate	273	31.6	32.4	27.7–37.4
High	84	9.7	8.9	6.2–12.6
Not stated	321	37.2	36.4	30.5–42.7
**Education level**				
Low	430	49.8	51.6	47.0–56.1
Moderate	336	38.9	37.9	34.2–41.6
High	98	11.3	10.6	8.4–13.2

a The ‘Wave of recruitment’ variable was constructed to represent the number of times a Wave 3 respondent previously participated in the survey to account for potential differences in individuals’ responses between those who were newly recruited at Wave 3 compared with those who participated in one or more prior survey waves^26^.

**Table 2:** Smoking behaviors of a nationally representative sample of adults who smoke from the ITC EUREST-PLUS Spain Survey, Spain, 2021 (N=864)

*Smoking behavior*	*Unweighted n*	*Weighted %*	*95 % CI*
**Smoking status**			
Non-daily	37	4.4	3.1–6.1
Daily	827	95.6	93.9–96.9
**Any attempt to quit in the past year**			
No attempt	765	88.7	86.2–90.8
At least one	99	11.3	9.2–13.8
**Intention to quit (binary)**			
Not planning to quit	467	54.4	49.8–58.8
Planning to quit	380	45.6	41.2–50.2
**Intention to quit (nominal)**			
In the next month	35	4.7	3.2–6.7
In the next 6 months	66	8.3	6.2–11.0
Beyond 6 months	279	32.7	28.4–37.2
Not planning to quit	467	54.4	49.8–58.8
**Cigarettes smoked per day**			
1–10	432	49.9	46.5–53.4
11–20	373	42.8	39.2–46.4
≥21	59	7.3	5.8–9.0
***Smoking behavior***	***n***	***Mean***	***95 % CI***
**Heaviness of smoking index**			
Overall	864	2.13	2.02–2.24
Non-daily	37	0.23^a^	0.08–0.39
Daily	827	2.22	2.11–2.33

a Relative standard error >0.3 (high sampling variability), interpret with caution.

Table 3 shows the adjusted prevalence of adults who intend to quit smoking by sociodemographic characteristics, behavioral characteristics, and intentions to quit smoking. The adults who smoke with the following characteristics reported the highest percentage of quit intentions: females (48.7%), aged 25–39 years (49.4%), have high levels of income (56.0%) and education (53.2%), smoke less than daily (58.6%), reported at least one quit attempt in the past year (63.6%), worried (49.4%) or thinking that smoking has damaged their health (48.8%), regretting starting to smoke (51.7%), concerned about their health (50.7%), received quitting advice from health professionals (51.6%), and reported the presence of smoking restrictions in public places (56.1%) as reasons that would lead them to quit.

**Table 3:** Association between sociodemographic and behavioral characteristics and intentions to quit smoking among a nationally representative sample of adults who smoke, ITC EUREST-PLUS Spain Survey, Spain, 2021 (N=755)

*Characteristics*	*Adjusted prevalence*		*Adjusted prevalence ratio*	
	*Adj. %^b^*	*95% CI*	*APR ^c^*	*95 % CI*
**Wave of recruitment^a^**				
Wave 1 (2016)	40.5	33.8–47.5	0.73	0.61–0.88
Wave 2 (2018)	39.7	31.8–48.2	0.72	0.58–0.89
Wave 3 (2021)	55.2	49.4–60.8	1.00	
**Sex**				
Male	46.0	40.1–52.0	0.95	0.82–1.10
Female	48.7	42.9–54.5	1.00	
**Age (years)**				
18–24	45.1	31.9–59.1	0.96	0.70–1.31
25–39	49.4	42.1–56.8	1.05	0.86–1.28
40–54	46.4	40.3–52.5	0.99	0.82–1.19
≥55	47.1	39.8–54.4	1.00	
**Income level**				
Not stated	50.7	44.5–56.9	1.26	0.95–1.68
High	56.0	45.3–66.1	**1.39**	**1.01–1.92**
Moderate	46.4	40.3–52.7	1.15	0.84–1.58
Low	40.2	29.8–51.6	1.00	
**Education level**				
High	53.2	43.1–63.0	1.44	0.84–2.45
Moderate	48.1	41.3–55.0	1.12	0.76–1.65
Low	45.7	39.9–51.6	1.00	
**Smoking status**				
Daily	46.9	42.2–51.6	0.80	0.63–1.02
Non-daily	58.6	43.7–72.0	1.00	
**Any attempts to quit in the past year**				
At least one	63.6	53.3–72.8	**1.41**	**1.16–1.71**
No attempt	45.1	39.9–50.4	1.00	
**Worried smoking will damage one's health**				
A little or more	49.4	44.2–54.6	**1.52**	**1.05–2.20**
Not at all	32.4	22.3–44.5	1.00	
**Extent smoking has damaged health**				
Just a little or more	48.8	43.9–53.8	1.13	0.94–1.37
Not at all	43.2	35.2–51.5	1.00	
**Regret over starting to smoke**				
Agree	51.7	46.2–57.1	**1.25**	**1.03–1.52**
Disagree	27.3	18.9–37.8	**0.66**	**0.46–0.95**
Neutral	41.3	33.4–49.7	1.00	
**Reason to quit: concern for health**				
Somewhat or more	50.7	45.4–56.0	**1.46**	**1.17–1.82**
Not at all	34.8	27.4–43.0	1.00	
**Reason to quit: advice from health care professional**				
Somewhat or more	51.6	43.9–59.2	1.16	0.96–1.39
Not at all	44.5	39.0–50.2	1.00	
**Reason to quit: restrictions in public places**				
Somewhat or more	56.1	47.1–64.8	**1.28**	**1.06–1.54**
Not at all	43.9	38.7–49.2	1.00	

a The ‘Wave of recruitment’ variable was constructed to represent the number of times a Wave 3 respondent previously participated in the survey to account for potential differences in individuals’ responses between those who were newly recruited at Wave 3 compared with those who participated in one or more prior survey waves26.

b Adj. %: adjusted percentage, or predictive margins, from a weighted Poisson regression model examining the association between sociodemographic and behavioral characteristics and intentions to quit smoking.

c APR: adjusted prevalence ratio estimated from a weighted Poisson regression model examining the association between sociodemographic and behavioral characteristics and intentions to quit smoking. Intentions to quit smoking (outcome variable): No intentions to quit smoking (0) versus Any intentions to quit smoking (1).

Several factors were associated with quit intentions, including , having high income level (adjusted prevalence ratio, APR=1.39; 95% CI:1.01–1.92), having at least one quit attempt in the past year (APR=1.41; 95% CI: 1.16–1.71), worrying that smoking will damage one’s health (APR=1.52; 95% CI: 1.05–2.20), regretting starting to smoke (agree: APR=1.25; 95% CI: 1.03–1.52 and disagree: APR=0.66; 95% CI: 0.46–0.95 compared to having a neutral opinion), being concerned about one’s health (APR=1.46; 95% CI: 1.17–1.82), and existence of smoking restrictions in public places (APR=1.28; 95% CI: 1.06–1.54).

## DISCUSSION

In 2021, less than half of adults who smoke in Spain intended to quit smoking and only 13.0% planned to quit in the next 6 months. The percentage of adults who smoke and who plan to quit in the next 6 months is lower than in other high- and middle-income countries surveyed by the ITC Project, including Brazil (48% in 2017), Malaysia (31% in 2014), Canada (43% in 2018), Australia (42% in 2018), the Netherlands (36% in 2017), South Korea (32% in 2016), France (33% in 2012), the United States (33% in 2018), China (28% in 2015), and Mexico (24% in 2015), but is higher than Japan (12% in 2019) and Germany (10% in 2018)^28^. Moreover, only 11.3% reported making any attempt to quit in the past year. Again, this was low compared to other ITC countries: Canada (44.5% in 2018), United States (31.7% in 2018), England (28.5% in 2018), and Australia (45.2% in 2018)^29^.

In this study, six factors were associated with intending to quit smoking: having a high income level, reporting at least one quit attempt in the past year, worrying that smoking will damage one’s health, regretting starting to smoke, being concerned about one’s health, and the existence of smoking restrictions in public places.

The first of these factors – high income – is consistent with other studies among adults who smoke^9,30^ and is worthy of further comment. Having more income increases financial accessibility to a variety of cessation products beyond what the government subsidizes; this may encourage quit intentions among adults who smoke. This finding highlights the lower rates of cessation and lower quit intentions that have been reported in other countries^30^. There is need for policy makers in Spain to address this disparity so that those who wish to quit have access to affordable cessation products of their choice. In Spain, smoking cessation treatments have only recently been offered to people who smoke and currently only one drug is subsidized^31^. The low support for cessation in Spain has been noted by the Tobacco Control Scale (TCS): Spain’s score assigned to cessation treatment provision has been low; however, there has been an improvement since 2019 (score in 2019: 5/10 points; score in 2021: 8/10 points)^32^. Further increases in support for cessation will lead to greater increases in quit intentions among those who smoke in Spain. This will lead to greater rates of quit attempts and successful quitting, ultimately leading to public health benefits that are known to be among the most cost-effective disease prevention measures^33^.

The finding that quit attempts in the past year were associated with quit intentions was also consistent with previous studies^11-15^. This is not surprising because adults who smoke often need multiple attempts to quit^34^. This finding highlights the need for legislation that encourages and supports people who smoke in their attempts to quit.

The finding that worrying about tobacco-related health effects or having any health concern was associated with quit intentions is consistent with other studies^11-15^. Additionally, health-related motivations are likely to have increased due to the COVID-19 pandemic that was ongoing when this study was conducted. During the same time period, there were several health education campaigns and the government implemented smoking restrictions in outdoor settings^35^. The study results highlight the importance of educating the public regarding tobacco-related harms, for example, using regular mass media campaigns, encouraging quitting, and implementing strong health warnings.

Consistent with other studies^16^, regretting starting to smoke serves as a powerful emotional motivator for future behavior change. Thus, anti-smoking campaigns need to integrate approaches that not only rely on rational explanations, but also appeal to the emotions of people who smoke (e.g. depicting negative social norms towards smoking) to effectively elicit regret and induce quit intentions^16^.

Our findings show that smoking restrictions in public places were associated with quit intentions, which is consistent with other studies. This finding is not surprising because during the COVID-19 pandemic, Spanish health authorities extended smoking restrictions outdoors, when a safe distance could not be maintained^35^. This emphasizes the importance of enforcing smoking restrictions in public places. Recently, the Spanish government announced that it will introduce additional tobacco control legislation, such as smoke-free terraces in hospitality venues^36^. This is a window of opportunity to advance this legislation in order to protect bystanders and workers from secondhand smoke. In addition, it is an opportunity to increase quit intentions^31^.

### Strengths and limitations

The limitations of this study include the use of cross-sectional data, which limits the ability to determine causality; and the reliance on self-reports, which can result in recall bias. Nevertheless, our results are consistent with other studies, showing similar factors that have been associated with quit intentions. Another limitation is the timing of our study, which was conducted during the COVID-19 pandemic. The government implemented several health education campaigns and smoking restrictions in outdoor settings that may have influenced the factors assessed. The two strengths of this study are the use of population-based data and standardized measures associated with quit intentions, which have been used in 30 countries participating in the ITC Project, allowing comparisons to be made across countries.

## CONCLUSIONS

In 2021, only 13% of adults from Spain who smoke reported that they intended to quit in the next 6 months. Factors associated with quitting were high income, having at least one quit attempt in the past year, worrying about health damage from smoking, regretting starting to smoke, having health concerns, and smoking restrictions in public places. There is a need for comprehensive programs that consider these factors and increase support for smoking cessation efforts among adults who smoke in Spain. Additionally, the implementation of other tobacco control measures can also serve to increase intentions to quit and quit attempts, and thus cessation should be seen in the broader context of tobacco control.

## Supplementary Material



## Data Availability

The data supporting this research are available from the authors on reasonable request.
